# Left-Sided Patent Ductus Arteriosus in a Right-Sided Aortic Arch

**DOI:** 10.1155/2014/896071

**Published:** 2014-11-17

**Authors:** Ming-Yen Ng, Paaladinesh Thavendiranathan, Andrew Michael Crean, Qin Li, Djeven Parameshvara Deva

**Affiliations:** ^1^Department of Medical Imaging, Toronto General Hospital, Toronto, ON, Canada M5G 2C4; ^2^Department of Diagnostic Radiology, The University of Hong Kong, Hong Kong; ^3^Division of Cardiology, Toronto General Hospital, Toronto, ON, Canada M5G 2C4; ^4^Department of Medical Imaging, St. Michael's Hospital, Toronto, ON, Canada M5B 1W8

## Abstract

We present a 31-year-old female with repaired tetralogy of Fallot (TOF) and right-sided aortic arch (RAA) with left-sided patent ductus arteriosus (PDA) originating from the left brachiocephalic artery. This is a rare finding but most common site for a PDA in TOF and a RAA. To the best of our knowledge, this is the first demonstration of this rare finding on MRI in the literature.

## 1. Introduction

We present a 31-year-old female with repaired tetralogy of Fallot (TOF) (transannular patch repair of the right ventricular outflow tract) and right-sided aortic arch with left-sided patent ductus arteriosus (PDA) originating from the left brachiocephalic artery, which is a rare finding, but most common site for a PDA in individuals with TOF and a right-sided aortic arch. To the best of our knowledge there are no other cases in the literature demonstrated on MRI.

## 2. Case Report

**Figure 1:**
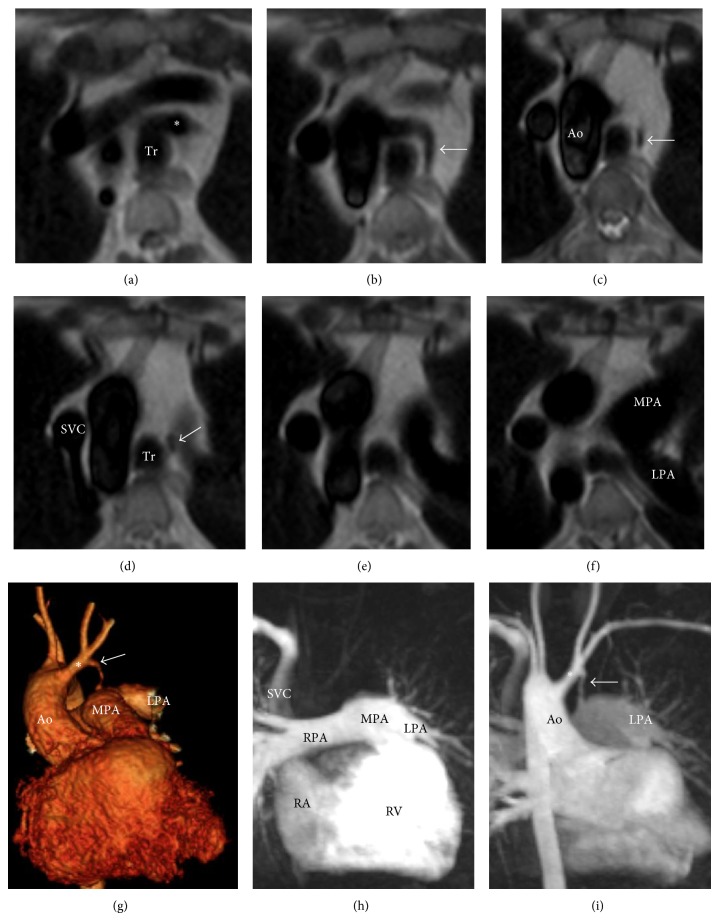
Axial half-Fourier acquisition single shot turbo spin echo images (images (a)–(f)) show the PDA (white arrow) coming off the left brachiocephalic artery and draining into the distal pulmonary trunk. 3D volume-rendered image (image (g), see Supplementary Video 1 in Supplementary Material available online at http://dx.doi.org/10.1155/2014/896071) demonstrating the PDA originating from the left brachiocephalic artery and draining into the distal pulmonary trunk. Maximum intensity projection of the time-resolved MRA (images (h) and (i), Supplementary Video 2), the PDA is not visible during the pulmonary arterial phase (h) but fills once contrast enters the right sided aortic arch during the systemic arterial phase ((i), arrow). Ao: aorta, ∗: left brachiocephalic artery, LPA: left pulmonary artery, RPA: right pulmonary artery, SVC: superior vena cava, Tr: trachea, MPA: main pulmonary artery, RA: right atrium, and RV: right ventricle.

The patient underwent cardiac magnetic resonance imaging (MRI) as part of investigations prior to pregnancy but was otherwise asymptomatic. MRI demonstrated a right-sided aortic arch with mirror image branching and a tubular structure connecting the left brachiocephalic artery to the distal pulmonary trunk (see Figure 1). The magnetic resonance angiogram (MRA) demonstrated blood flow through the tubular structure but the pulmonary-systemic stroke volume ratio (Qp : Qs ratio) was 1 : 1. Therefore, there was no significant shunt and no intervention was required. Previous imaging was done in a different institution more than twenty years previously and was not available for comparison. The differential diagnosis for this appearance was a modified Blalock-Taussig shunt but this was ruled out based on surgical notes. The PDA position is consistent with Edward's developmental model of the aortic arch.

## 3. Discussion

In Edward's developmental model of the aortic arch the development of the right-sided aortic arch with mirror image branching occurs due to involution of the dorsal segment of the left arch between the left subclavian artery and descending aorta [1–3]. The right ductus arteriosus also undergoes involution and the remaining left ductus arteriosus usually originates from the left brachiocephalic artery or subclavian artery. This configuration may form a vascular ring with associated symptoms of dysphagia (though this was not the case in our patient). While this is the most common anatomical arrangement in tetralogy of Fallot with a right aortic arch, it is still an extremely rare observation in adult clinical practice.

Other documented positions of the PDA in patients with tetralogy of Fallot include origins from an aberrant left subclavian artery in a right aortic arch, from a diverticulum which also gives off the aberrant left subclavian artery, and from the left common carotid artery in the context of aberrant left subclavian artery, and, rarely, there can be a right PDA connected to the right pulmonary artery. The PDA can arise from the right subclavian artery in a left aortic arch or from a diverticulum when in conjunction with an aberrant right subclavian [4].

## Supplementary Material

Supplementary Video 1: 3D volume-rendered cine of the whole heart showing the PDA connecting the left brachiocephalic artery to the distal pulmonary trunk.
Supplementary Video 2: Maximum intensity projection time resolved MRA showing the PDA enhancing with contrast during the arterial phase. The PDA originates from the left brachiocephalic artery and drains into the distal pulmonary trunk.
